# Persistence and geographic availability of Medicare Part D coverage for non-diabetes GLP-1 therapies, 2024-2026

**DOI:** 10.1093/haschl/qxag299

**Published:** 2026-10-25

**Authors:** Eunhae Shin, Alice Chen

**Affiliations:** Department of Health Policy and Management, University of Georgia, Athens, GA 30602, United States; Sol Price School of Public Policy and Leonard D. Schaeffer Institute for Public Policy & Government Service, University of Southern California, Los Angeles, CA 90089, United States

**Keywords:** Medicare, formularies, access to care, GLP-1 receptor agonists, semaglutide, tirzepatide

## Introduction

Glucagon-like peptide-1 (GLP-1) receptor agonists approved for diabetes are widely covered by Medicare Part D, but Part D is statutorily prohibited from covering drugs when used for weight loss.^1^ Beginning in 2024, however, two GLP-1s initially approved for weight management—semaglutide (Wegovy) and tirzepatide (Zepbound)—gained non-weight-loss indications eligible for Part D coverage, including cardiovascular risk reduction, metabolic dysfunction-associated steatohepatitis with fibrosis, and obstructive sleep apnea (Table S1).

Prior research found that Part D coverage was widespread for GLP-1 s for diabetes but rare for non-diabetes indications through 2025.^2^ Whether plans subsequently expanded or sustained such coverage, what factors might explain coverage withdrawal, and whether it reached areas with greater disease burden remain unknown.

We examined Medicare Advantage prescription drug (MA-PD) and stand-alone prescription drug plans (PDPs) from 2024 through 2026 to estimate changes and persistence in Wegovy and Zepbound coverage, potential explanations for coverage withdrawal, and geographic availability relative to county disease prevalence.

## Methods

We used Centers for Medicare & Medicaid Services (CMS) Prescription Drug Plan Formulary Public Use Files released in June 2024, 2025, and 2026. A plan was classified as listing a GLP-1 if its formulary included ≥1 National Drug Code for Wegovy or Zepbound (Table S2). The unit of analysis was the contract-plan. Enrollment-weighted estimates used June CMS plan-level enrollment files. Plans were linked across years by contract and plan identifier and classified as retaining coverage, operating without coverage, or terminated. To examine potential explanations for coverage changes, we tested whether GLP-1 coverage was associated with changes in plan enrollment or enrollee cardiometabolic burden. We also considered whether dropped coverage was associated with broader formulary retrenchment or greater likelihood of overall plan termination (Supplemental Methods).

County availability was measured using the CMS Landscape plan-county files. County prevalence of adult obesity and coronary heart disease came from the Centers for Disease Control and Prevention PLACES 2024 release. We estimated county-level associations between GLP-1 coverage and prevalence using linear probability models with state fixed effects and standard errors clustered by state.

Analyses used Stata version 19.5 (StataCorp, College Station, TX). This study used publicly available data and did not require institutional review board review.

## Results

Non-diabetes GLP-1 coverage remained rare and confined to MA-PDs: 54 of 4925 (1.1%) listed a GLP-1 in 2024, and 89 of 4768 (1.9%) in 2025, falling to 44 of 4707 (0.9%) in 2026 (Table S3). No PDP listed either drug. Weighted by enrollment, 1.3%, 1.7%, and 0.7% of MA-PD beneficiaries were in plans listing a GLP-1. The listing was unchanged in the July 2026 file, released after the Medicare GLP-1 Bridge began covering GLP-1 s for weight management outside Part D (Table S4).^3^

Coverage was rarely sustained. Of plans listed in in 2024 and 2025, only 52% and 19% retained coverage the next year (42% and 16% of their enrollment; Figure 1A). Among plans that did not continue coverage, the benefit was either dropped or the plan itself was terminated. Plan terminations accounted for 22% and 47% of listing plans (17% and 31% of their enrollment) by 2025 and 2026, respectively.

**Figure 1.**
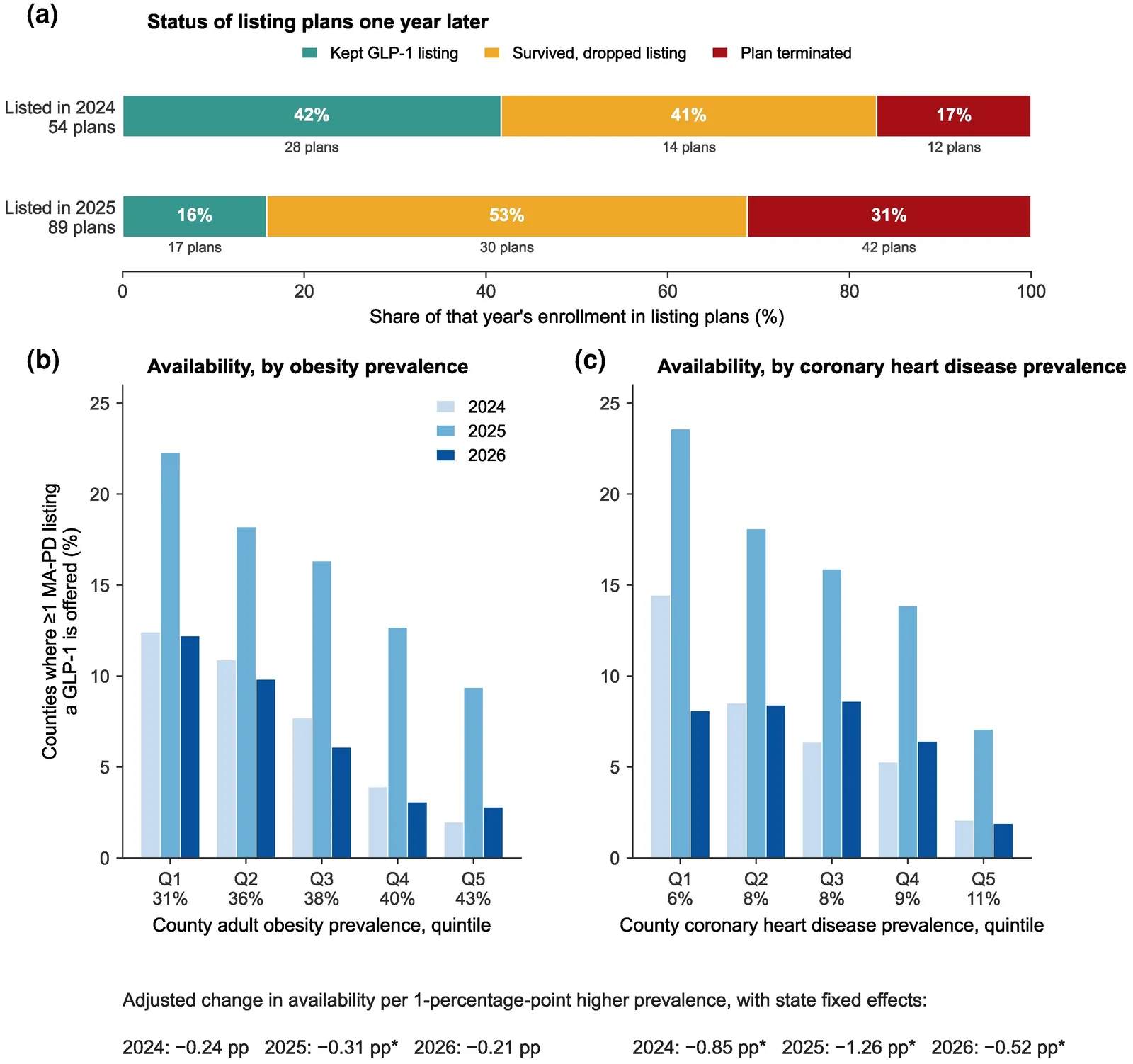
Persistence of GLP-1 coverage and geographic availability relative to disease prevalence, 2024-2026. *Abbreviations:* BRFSS, Behavioral Risk Factor Surveillance System; CDC, Centers for Disease Control and Prevention; GLP-1, glucagon-like peptide-1; MA-PD, Medicare Advantage prescription drug plan. In Panel A, each MA-PD listing a GLP-1 in the stated year (54 plans with 365 391 enrollees in 2024; 89 plans with 478 758 enrollees in 2025) is followed into the subsequent year. No stand-alone prescription drug plans listed GLP-1s during 2024-2026. In Panels B and C, counties are grouped into quintiles of adult crude disease prevalence from the CDC PLACES 2024 release (BRFSS 2022), with mean prevalence shown for each quintile. The analysis includes counties in the 50 states and District of Columbia with at least 1 MA-PD offered: 3037 in 2024, 3023 in 2025, and 3025 in 2026, of 3144 counties covered by PLACES. Coefficients shown below Panels B and C are from separate year-specific linear probability models; the outcome is availability of at least 1 MA-PD listing a GLP-1 in the county, and the exposure is continuous county prevalence (percentage points). Models include state fixed effects, with standard errors clustered by state. * *P* < 0.05.

Observable changes in enrollee composition or formulary policy did not explain coverage withdrawal (Table S5). Compared with plan contracts that never covered GLP-1s, contracts initiating coverage in 2024, where most enrollees were in listing plans, experienced no differential change in the share of enrollees with diabetes (−0.4% points; *P* = 0.75) or cardiovascular disease (−0.1; *P* = 0.82), nor did they attract enrollment in subsequent years (−3.7%; *P* = 0.51). Plans that withdrew coverage also did not significantly reduce overall formulary breadth (−1.5%; *P* = 0.24) relative to same-county plans that never provided coverage. Although listing plans were more likely to terminate, this difference was attenuated after adjustment for plan characteristics.

Geographic availability expanded in 2025 before contracting in 2026 and was consistently lower in counties with greater disease burden (Figures 1B and 1C). In 2025, coverage was available in 22.3% of counties in the lowest obesity quintile versus 9.4% in the highest, and 23.6% versus 7.1% across coronary heart disease quintiles. With state fixed effects, each 1-percentage-point higher coronary heart disease prevalence was associated with 0.85-, 1.26-, and 0.52-percentage-point lower availability in 2024, 2025, and 2026 (all *P* < 0.05); associations with obesity were also negative in all 3 years, although significant only in 2025.

## Discussion

Despite three new FDA-approved non-weight-loss indications,^4^ Part D coverage of non-diabetes GLP-1s remained rare, exclusively in Medicare Advantage, and was rarely sustained. Coverage losses reflected both plan exit and withdrawal among continuing plans. Withdrawal appeared specific to GLP-1s rather than broader formulary retrenchment, as plans dropping coverage did not generally narrow their formularies relative to same-county competitors. We also found little evidence of realized adverse selection through increased enrollment or cardiometabolic burden. Although our analyses do not identify why continuing plans withdrew coverage, one possibility is greater plan liability for high-cost drugs following the Inflation Reduction Act's 2025 Part D redesign.^5^ The Bridge appears unlikely to explain withdrawal: 2026 withdrawals predated its July launch, with no further withdrawals that month.

Limited coverage was also poorly aligned with disease burden. Across all 3 years, GLP-1 coverage was consistently less available in counties with higher prevalence of coronary heart disease and was also negatively associated with obesity prevalence, although less consistently.

Limitations include reliance on formulary listings rather than dispensing data, which do not capture access through formulary exceptions or, for weight management, the Bridge; inability to observe indication at the point of sale; and use of ecological county prevalence. Nonetheless, following plans over time and across their service areas shows that expanding FDA-approved indications alone may not produce durable Medicare access or coverage aligned with geographic disease burden. The 2026 contraction is consistent with broader challenges facing voluntary coverage, including CMS's April 2026 decision to delay the Part D component of the voluntary Better Approaches to Lifestyle and Nutrition for Comprehensive Health (BALANCE) Model because of limited plan participation.^3^ Together, these findings raise questions about whether voluntary plan-based coverage can provide sustained and broadly available access as Medicare-eligible GLP-1 indications expand.^6^

## Supplementary Material

qxag299_Supplementary_Data
