# Supplementary material for: Persistence and geographic availability of Medicare Part D coverage for non-diabetes GLP-1 therapies, 2024-2026
Source: Health Aff Sch. 2026 Oct 25;4(11):qxag299. doi: 10.1093/haschl/qxag299 (PMC13732724; doi:10.1093/haschl/qxag299)
Supplement: Supplementary file 1 [file qxag299_supplementary_data.zip › GLP1_PartD_coverage_supplement_clean.docx]

# **SUPPLEMENT**

**Supplemental Methods**

*Data sources.* We used the Centers for Medicare & Medicaid Services (CMS) monthly Prescription Drug Plan Formulary, Pharmacy Network, and Pricing Information public-use files released in June of each year, corresponding to data dated June 30, 2024; June 12, 2025; and June 10, 2026. From each release, we used the plan information and basic drugs formulary files. The plan universe (one record per contract-plan), plan type, and Special Needs Plan status were derived from the plan information file. Overall Star Ratings came from the Landscape file, and plan enrollment from the Monthly Enrollment by Contract/Plan/State/County (CPSC) file (June of each year). CMS suppresses plan-county enrollment counts below 11. For plan-level totals, suppressed cells were assigned a value of 5. Among plans listing a GLP-1 this affects 789, 1,658, and 762 plan-county cells and accounts for 1.1%, 1.7%, and 1.8% of reported enrollment in 2024, 2025, and 2026, respectively. County-level analyses use the unimputed file.

*GLP-1 identification.* The US Food and Drug Administration (FDA)-approved indications for each agent are summarized in Table S1. A plan was classified as listing a GLP-1 if its basic formulary included at least 1 of 7 Wegovy or 12 Zepbound National Drug Codes (Table S2). Oral Wegovy was excluded because it became available only in 2026 and could not be examined longitudinally.

*Geographic analysis.* Geographic availability was measured using the CMS Landscape plan-county files, which identify every county in a plan's service area: the CY2024 Landscape Medicare Advantage and Special Needs Plan files, the CY2025 Landscape file released June 2025, and the CY2026 Landscape file released March 2026. County names were matched to Federal Information Processing Standards (FIPS) codes using the Census county file, matching 99.0% (2024), 99.1% (2025), and 99.3% (2026) of service-area records; unmatched records were predominantly Alaska boroughs and census areas. The analysis denominator was counties in which at least 1 Medicare Advantage prescription drug plan (MA-PD) was offered, because availability of GLP-1 coverage is undefined where no MA-PD operates: 3,037 (2024), 3,023 (2025), and 3,025 (2026) of the 3,144 counties covered by PLACES.

County crude prevalence of adult obesity and coronary heart disease came from the Centers for Disease Control and Prevention (CDC) PLACES 2024 release, which provides model-based county estimates using 2022 Behavioral Risk Factor Surveillance System data. PLACES does not cover Puerto Rico, which was excluded from county analyses. PLACES contains no measure of obstructive sleep apnea (its only sleep-related metric is short sleep duration) and none of metabolic dysfunction-associated steatohepatitis or chronic liver disease; therefore, prevalence specific to those indications could not be mapped.

Associations between county availability and prevalence were estimated using linear probability models with state fixed effects and standard errors clustered by state.

*Analyses of Potential Explanations for Coverage Withdrawal.* To assess realized adverse selection, we estimated the association between GLP-1 listing and plan enrollment using a plan-county-year panel with plan-county and county-year fixed effects and standard errors clustered by contract. Identification therefore came from within-plan-county changes in listing status relative to contemporaneous enrollment changes among other plans offered in the same county.

We also compared contract-level comorbidity burden using the CMS Medicare Advantage Healthcare Effectiveness Data and Information Set (HEDIS) public use files, which report the number of enrollees meeting each measure’s clinical criteria along with contract enrollment. Diabetes burden was measured using the denominators for Kidney Health Evaluation for Patients With Diabetes and Statin Use in Persons With Diabetes, and cardiovascular burden using the denominator for Statin Therapy for Patients With Cardiovascular Disease. These measures capture enrollee comorbidity and are distinct from the county disease prevalence used in the geographic analysis in Figure 1. None of the 36 measures reported in these files captures obesity or body mass index; therefore, enrollee obesity cannot be observed at the contract level, and diabetes and cardiovascular disease served as available cardiometabolic proxies for populations likely to seek GLP-1 coverage. CMS began publishing these counts in the 2024 release, so the panel spans measurement years 2023 and 2024 and does not permit assessment of pre-trends. Measurement year 2025 is not yet available, so the 2026 withdrawal could not be examined using these measures.

Listing status was defined at the contract level, the level at which HEDIS measures are reported. Although one-third of MA-PD contracts used more than 1 formulary, GLP-1 listing was uniform across a contract’s MA-PD plans in 59 of the 60 contract-years in which any plan listed, including all 10 contracts listing in 2024. Because HEDIS denominators also include employer group and MA-only enrollees, who are absent from formulary files, we conducted sensitivity analyses that weighted each contract’s listing status by its share of enrollment in listing plans or restricted to contracts in which this share was at least 50% (Table S5).

To assess whether GLP-1 withdrawal reflected more general benefit retrenchment, we compared year-over-year changes in formulary breadth, measured by the number of distinct RxNorm Concept Unique Identifiers (RxCUIs). Results are reported in Table S5.

All statistical analyses were conducted using Stata version 19.5 (StataCorp, College Station, Texas).

**Table S1. FDA-Approved Indications for Drugs Marketed for Chronic Weight Management, as of Plan Year 2026**

| **Drug** | **Obesity / overweight** | **Cardiovascular risk reduction** | **Obstructive sleep apnea** | **MASH with fibrosis**^a^ |
| --- | --- | --- | --- | --- |
| Wegovy (semaglutide) | Yes (2021) | Yes (Mar 8, 2024) | No | Yes (Aug 15, 2025) |
| Zepbound (tirzepatide) | Yes (Nov 2023) | No | Yes (Dec 20, 2024) | No |

^a^ MASH indicates metabolic dysfunction-associated steatohepatitis; accelerated approval for noncirrhotic MASH with moderate to advanced liver fibrosis.

The cardiovascular indication requires established cardiovascular disease with obesity or overweight, and the sleep apnea indication requires obesity, whereas the MASH indication does not require obesity.

**Table S2. National Drug Codes (NDCs) Used to Identify Wegovy and Zepbound**

| **Strength** | **NDC Code** |
| --- | --- |
| *Wegovy* | |
| 0.25 mg/0.5 mL | 0169-4525-14 |
| 0.5 mg/0.5 mL | 0169-4505-14 |
| 1 mg/0.5 mL | 0169-4501-14 |
| 1.7 mg/0.75 mL | 0169-4517-14 |
| 2.4 mg/0.75 mL | 0169-4524-14 |
| 0.25 mg/0.5 mL | 0169-4525-94 |
| 0.25 mg/0.5 mL | 50090-5824-0 |
| *Zepbound – pen* | |
| 2.5 mg/0.5 mL | 0002-2506-80 |
| 5 mg/0.5 mL | 0002-2495-80 |
| 7.5 mg/0.5 mL | 0002-2484-80 |
| 10 mg/0.5 mL | 0002-2471-80 |
| 12.5 mg/0.5 mL | 0002-2460-80 |
| 15 mg/0.5 mL | 0002-2457-80 |
| *Zepbound – vial* | |
| 2.5 mg/0.5 mL | 0002-0152-01 |
| 5 mg/0.5 mL | 0002-0243-01 |
| 7.5 mg/0.5 mL | 0002-1214-01 |
| 10 mg/0.5 mL | 0002-1340-01 |
| 12.5 mg/0.5 mL | 0002-1423-01 |
| 15 mg/0.5 mL | 0002-2002-01 |

**Table S3. Formulary Listing, County Availability, and Enrollment Composition of GLP-1 Coverage in Medicare Part D Plans, 2024–2026**

|  | **2024** | **2025** | **2026** |
| --- | --- | --- | --- |
| MA-PD plans, No. | 4,925 | 4,768 | 4,707 |
| Listing a GLP-1, No. (%) | 54 (1.1) | 89 (1.9) | 44 (0.9) |
| Stand-alone PDPs listing, No./Total | 0/719 | 0/474 | 0/367 |
| Beneficiaries in listing plans, No. | 365,391 | 478,758 | 209,258 |
| % of MA-PD enrollment | 1.3 | 1.7 | 0.7 |
| Among MA-PD listing plans, No. (%) |  |  |  |
| Semaglutide (Wegovy) | 54 (100) | 89 (100) | 44 (100) |
| Tirzepatide (Zepbound) | 0 (0) | 61 (69) | 26 (59) |
| Retained listing from prior year^a^ | — | 28 (52) | 17 (19) |
| Counties where ≥1 listing plan was offered, No. | 223 | 476 | 204 |
| Losing all coverage by the next year, No.(%)^b^ | 24 (10.8) | 289 (60.7) | — |
| MA-PD enrollees in those counties, No.^b^ | 990,338 | 3,739,426 | — |

***Abbreviations:*** GLP-1, glucagon-like peptide-1; MA-PD, Medicare Advantage prescription drug plan; PDP, stand-alone prescription drug plan.

^a^ Among plans listing in the prior year (denominators 54 for 2025, 89 for 2026).

^b^ Loss is measured against the following year and therefore cannot be computed for 2026; enrollee counts reflect MA-PD enrollment in the following year among counties that lost all coverage.

**Table S4. GLP-1 Listing in Medicare Advantage Prescription Drug Plans Before and After the Medicare GLP-1 Bridge, June vs July Formulary Files, 2024–2026**

|  | **2024** | **2025** | **2026** |
| --- | --- | --- | --- |
| MA-PD plans listing a GLP-1, No. (%) |  |  |  |
| June file | 54 (1.1) | 89 (1.9) | 44 (0.9) |
| July file | 54 (1.1) | 98 (2.1) | 44 (0.9) |
| Added, June to July, No. | 0 | 9 | 0 |
| Dropped, June to July, No. | 0 | 0 | 0 |
| Beneficiaries in listing plans, No. (% of MA-PD enrollment)^a^ |  |  |  |
| June file | 365,391 (1.3) | 478,758 (1.7) | 209,258 (0.7) |
| July file | 365,391 (1.3) | 511,983 (1.8) | 209,258 (0.7) |
| Retained listing in the following year, No./total (%)^b^ |  |  |  |
| June to June | 28/54 (52) | 17/89 (19) | — |
| July to July | 28/54 (52) | 26/98 (27) | — |

***Abbreviations:*** CMS, Centers for Medicare & Medicaid Services; GLP-1, glucagon-like peptide-1; MA-PD, Medicare Advantage prescription drug plan.

June and July files are the CMS monthly formulary public use files dated June 30 and July 31 of each year; the Medicare GLP-1 Bridge was announced December 23, 2025, and launched July 1, 2026. All 2026 withdrawals were present in January 2026 formularies, and no plan listing a GLP-1 in January stopped listing it by July.

^a^ Enrollment is from June of the same year and held fixed across June and July formulary files.

^b^ Plans terminated in the following year are counted as not retained; not computable for 2026.

**Table S5. Tests of Competing Explanations for Withdrawal of GLP-1 Coverage**

| **Explanation tested** | **Specification** | **Estimate (95% CI)** | **P value** |
| --- | --- | --- | --- |
| **A. Adverse Enrollee Composition** | | | |
| Enrollee diabetes burden rose in covering plans | Difference-in-differences on HEDIS measurement years 2023 and 2024: contracts that listed in plan year 2024 vs contracts that never listed; outcome is the share of enrollees meeting HEDIS diabetes criteria; contract and year fixed effects | −0.4 pp (−2.6 to 1.8) | .75 |
|  | Same, listing status weighted by share of contract enrollment in listing plans | −0.7 pp (−3.2 to 1.7) | .56 |
|  | Same, restricted to contracts with ≥50% of enrollment in listing plans | −0.8 pp (−2.9 to 1.3) | .46 |
| Enrollee cardiovascular burden rose in covering plans | Same design and years: outcome uses HEDIS cardiovascular disease criteria | −0.1 pp (−0.8 to 0.6) | .82 |
|  | Same, listing status weighted by share of contract enrollment in listing plans | −0.1 pp (−1.0 to 0.7) | .75 |
|  | Same, restricted to contracts with ≥50% of enrollment in listing plans | −0.2 pp (−0.9 to 0.5) | .64 |
| Listing attracted enrollment (realized adverse selection) | Plan-county-year panel pooling 2024–2026: outcome is ln(plan-county enrollment); plan-county and county-year fixed effects | −3.7% (−14.9% to 7.4%) | .51 |
| **B. Broad Changes in Formulary Design** | | | |
| Withdrawal was part of general formulary retrenchment | Plan-county-year panel pooling the 2024–2025 and 2025–2026 transitions: outcome is the change in distinct RxCUIs for plans dropping a GLP-1 vs plans that never listed; county-year fixed effects | −1.5% (−4.0% to 1.0%) | .24 |
| **C. Termination of Plan** | | | |
| Listing plans were more likely to be terminated, 2025–2026 | Linear probability model on plan-county observations in 2025: outcome is the plan’s absence from the 2026 file; county fixed effects | 24.5 pp (3.0 to 46.1) | .025 |
|  | Same, adding ln(enrollment), SNP status, and Overall Star Rating | 15.8 pp (−5.9 to 37.5) | .15 |

***Abbreviations:*** CI, confidence interval; GLP-1, glucagon-like peptide-1; HEDIS, Healthcare Effectiveness Data and Information Set; pp, percentage points; RxCUI, RxNorm Concept Unique Identifier; SNP, Special Needs Plan.

Standard errors are clustered at the contract level throughout.
